# Impacts of Multi‐Land Use Decisions on Temperate Forest Habitat Quality in the Changbai Mountain Region, Northeast China

**DOI:** 10.1002/ece3.71123

**Published:** 2025-03-27

**Authors:** Li Liu, Wen J. Wang, Lei Wang, Yu Cong, Haitao Wu

**Affiliations:** ^1^ Northeast Institute of Geography and Agroecology, Chinese Academy of Sciences Changchun China; ^2^ University of Chinese Academy of Sciences Beijing China

**Keywords:** ecological program, forest management, habitat fragmentation, habitat loss, land use change, temperate forests

## Abstract

Human‐driven land use changes significantly contribute to habitat loss and fragmentation in temperate forests, prompting the implementation of ecological conservation programs. However, these efforts may be undermined by the competing demands of ecological conservation and economic development. This study assessed changes in temperate forest habitat quality and the relative contribution of competing land use decisions (ecological programs, cropland expansion, and urbanization) to these changes in the Changbai Mountain region, Northeast China from 1990 to 2050. Our results revealed a region‐wide decline (−20.77%) in habitat quality over the past 30 years, with projected improvements (+14.64%) under the future scenario, albeit with considerable regional variations. Ecological programs contributed to long‐term habitat improvements by preserving and expanding forest cover. However, cropland expansion and urbanization through forest conversion were identified as the primary drivers of habitat quality degradation, leading to both direct habitat loss and indirect negative effects on the quality of the remaining habitat. Our findings offer valuable insights into the effectiveness of ecological programs and the trade‐offs posed by economic pressures, highlighting the need for integrated land use strategies that balance ecological and socio‐economic objectives in temperate forest management.

## Graphical abstract

Impacts of multi‐land use decisions on forest habitat quality were assessed. Cropland expansion and urbanization undermined efforts of ecological programs. Future forest management should consider trade‐offs of multi‐land use decisions.

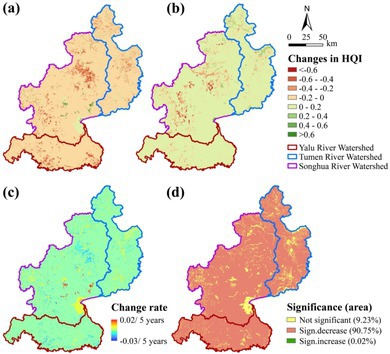

## 1 Introduction

Temperate forests are globally important ecosystems, serving as critical habitats for unique species and providing essential ecosystem services (Landuyt et al. 2019; Santini et al. 2019; Zhao et al. 2024). However, they are among the most heavily disturbed forest ecosystems due to extensive human activities (Lin et al. 2024; Liu et al. 2023; McDowell et al. 2020). Habitat loss and fragmentation, primarily driven by anthropogenic land use changes such as deforestation, urbanization, and cropland expansion (Ding et al. 2024; Grantham et al. 2020; Mondal et al. 2021), are among the most pressing threats to biodiversity in temperate forests (Brooks et al. 2002; Davison et al. 2021; Fardila et al. 2017; Kuipers et al. 2021). These disturbances significantly alter ecosystem composition and structure and disrupt key ecological processes (e.g., water regulation and species interactions), thereby accelerating biodiversity loss and diminishing the capacity of temperate forests to sustain their ecological functions and services (Fischer et al. 2013; Jo et al. 2024; Rodriguez‐Echeverry et al. 2018; Stritih et al. 2024).

To mitigate the adverse impacts of human disturbances (Li, Guo et al. 2023; Navarrete et al. 2023), various ecological programs, such as reduced deforestation, afforestation, and sustainable forest management, have been implemented globally (Deng et al. 2017; Liu et al. 2024; Zeng et al. 2020). For example, global tree cover increased by 2.24 million km^2^ between 1982 and 2016, with 60% of the gains attributed to direct human activities (Song et al. 2018). Despite these efforts, ongoing land use changes driven by economic demands, such as cropland expansion and urbanization, often undermine these conservation and restoration initiatives (Hossain et al. 2022; Roy et al. 2024; Winberg et al. 2023). Therefore, assessing the impacts of competing land use decisions on temperate forest habitat is essential for informing ecosystem management decisions and biodiversity protection.

China has implemented several ecological restoration programs since the late 1970s, covering approximately 65% of the country's land area, with tree planting initiatives accounting for 48.55% of all efforts (Bryan et al. 2018; Chen et al. 2022; Lu et al. 2018). Two of the most prominent programs, the Natural Forest Conservation Program (NFCP) and the Grain for Green Program (GGP), initiated in 1999 and 2000, represent the largest forest restoration initiatives nationwide (Liu et al. 2008; Zhang et al. 2024). Recent studies suggest that these programs have successfully increased forest cover, enhanced carbon sequestration, controlled soil erosion, and improved human well‐being (Cheng et al. 2024; Huang et al. 2019). For example, China contributed 25% of global greening, primarily driven by large‐scale tree plantations in northern temperate forests (Chen et al. 2019; Liu et al. 2024); these afforestation efforts also significantly enhanced terrestrial carbon sinks through increased carbon sequestration in plant biomass and soils (Chen et al. 2021; Hong et al. 2023; Yu et al. 2022). Nevertheless, cropland expansion into forests and grasslands between 2000 and 2015 resulted in a 113.8% reduction in wildlife habitats, a 29.0% decrease in carbon sequestration, and a 10.2% decline in soil retention at the national level (Kong et al. 2023). Despite these developments, the cumulative effects of competing land use decisions on habitat quality and the effectiveness of ecological programs remain largely unknown.

Habitat quality is commonly used as a surrogate for ecosystem health and biodiversity (Zhang et al. 2024). Recent studies on habitat quality often focus on specific species, primarily through traditional field investigations of biodiversity and habitats (Mondragón‐Botero et al. 2024; Regolin et al. 2021; Wen et al. 2024). Bioindicator species are commonly used to assess habitat quality by examining their biological characteristics, abundance, and survival, typically at the stand scale (Bace et al. 2023; Ikauniece et al. 2012; Tobisch et al. 2023). However, these studies often lack long‐term and continuous spatial distribution data on species occurrence to assess the spatio‐temporal dynamics of habitat quality (Sun et al. 2019; Tang et al. 2020). Some studies assessed habitat quality by integrating ecological indicators and remote sensing data, but they are unable to incorporate the ecological processes driving habitat change (Soto et al. 2017). Although ecological niche models (e.g., MaxEnt) and field data are commonly used to predict biodiversity distribution (Thulasi et al. 2024; Vergara et al. 2019), acquiring species information at large scales remains challenging. Additionally, some studies demonstrated that the spatial pattern of habitat changes can directly or indirectly affect habitat size, diversity, and connectivity, which in turn influence habitat quality and biodiversity (Diniz et al. 2022; Sun et al. 2019; Zhang et al. 2024). Therefore, it is essential to integrate spatial information at long time scales and large spatial extents for assessing forest habitat quality dynamics, which better informs the effectiveness of ecological programs and the effects of competing land uses on temperate forest habitat quality.

This study assessed the impacts of land use changes driven by ecological programs (the NFCP and GGP) and economic demands (cropland expansion and urbanization) on forest habitat quality in the Changbai Mountain region (CBMR) from 1990 to 2050. The CBMR, one of the largest forested areas in China and a focal region for the NFCP and GGP, encompasses nearly all vegetation types from the temperate zone to the polar zone, which are crucial for biodiversity conservation and national ecological security (Jin et al. 2024). However, as one of the vital food‐producing regions and the earliest industrial base of China, the CBMR has experienced intensive deforestation and degradation over the past decades due to urbanization and cropland expansion (Zhang et al. 2022; Zheng et al. 1997). The objectives of this paper were to assess: (1) land use changes in the CBMR from 1990 to 2050 in response to competing land use decisions, (2) the spatiotemporal changes in forest habitat quality, and (3) the effects of multi‐land use decisions on these habitat quality changes.

## 2 Materials and Methods

### 2.1 Study Area

**FIGURE 1:**
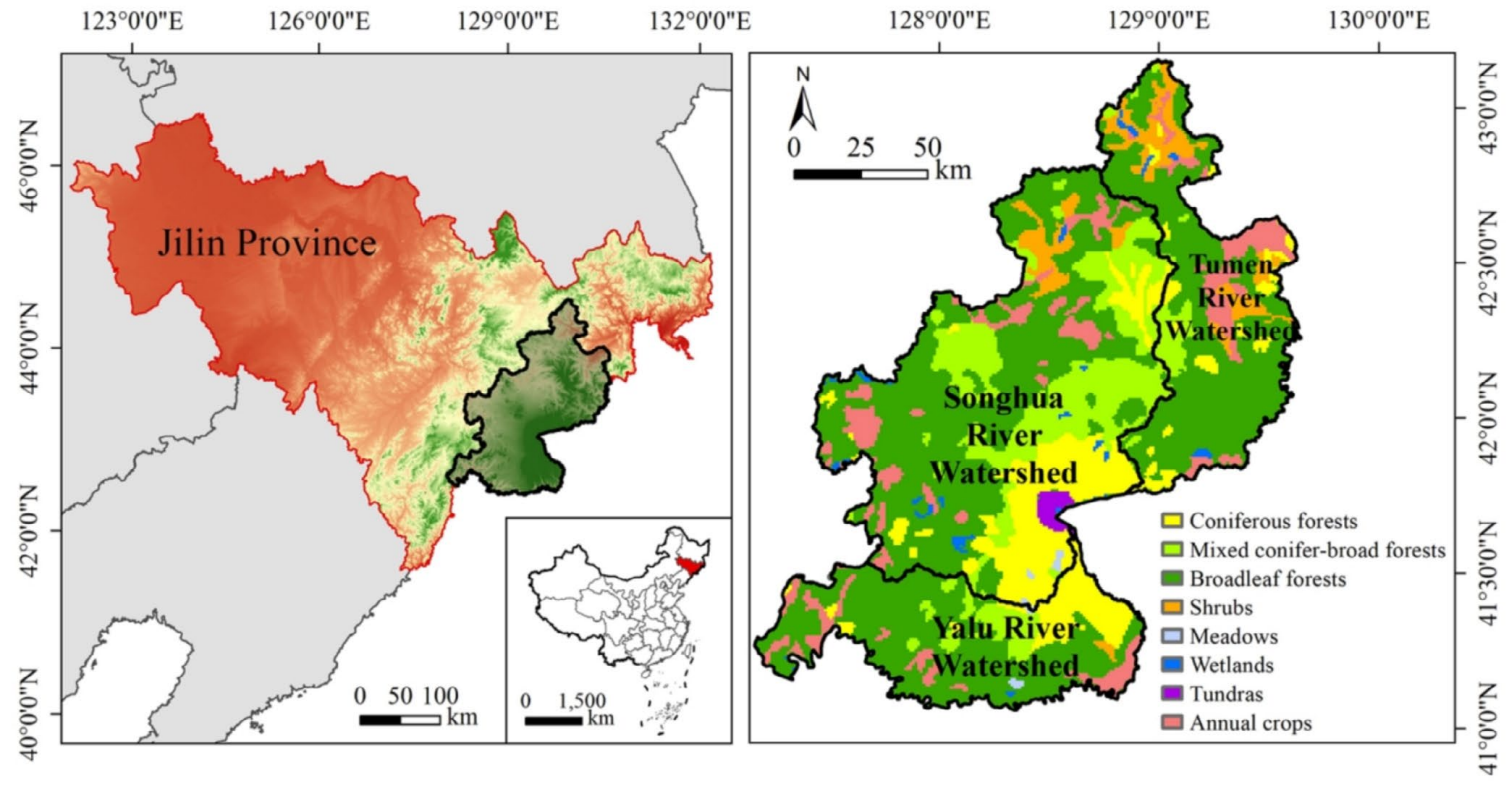
Location (left) and vegetation types (right) of the Changbai Mountain region (CBMR).

Our study area was located in the Changbai Mountain region (CBMR), Northeast China (126.49° E to 129.64° E, 41.18° N to 43.70° N) (Figure 1). It includes the Changbai Nature Reserve and the core region of the Changbai Mountains. The elevation of the CBMR is low in the north and high in the south, reaching a maximum of 2670 m and a minimum of 273 m. The area of the CBMR is approximately 24,200 km^2^, encompassing three watersheds: the Songhua River Watershed (53.52%), the Yalu River Watershed (24.54%), and the Tumen River Watershed (21.94%). The CBMR has a temperate continental monsoonal climate, with an average annual temperature of 4.38°C and an average annual precipitation of 741.85 mm, which varies significantly with altitude. Most of the CBMR is forested, predominantly with temperate broadleaf forests, followed by conifer‐broadleaved mixed forests. The CBMR is an inactive volcanic region with rich forest resources and biodiversity, providing crucial ecosystem services, such as species habitats (Wang et al. 2022). These forest habitats support the survival and reproduction of endangered species such as the Scaly‐sided Merganser ( *Mergus squamatus* ) and endemic species such as the Northeastern China Salamander ( *Hynobius leechii* ) (Han et al. 2024; Yang et al. 2024).

Over the past decades, land use in the CBMR experienced significant changes driven by increasing food demands, economic growth, and environmental protection initiatives. Since the 1950s, human activities such as deforestation, reclamation, and urbanization converted large areas into construction land or cropland (Ren et al. 2024). Extensive forest loss and rapid cropland expansion prompted the implementation of the NFCP and GGP in 1999 and 2000, respectively. The NFCP protects natural forests through logging bans and reforestation efforts, which have been implemented on 197.73 million ha of natural forests in China by 2019; the GGP undertakes large‐scale afforestation of croplands with slopes exceeding 20°, which has converted approximately 502 million ha of croplands into forests by the middle of 2019 (Zhou et al. 2022). Nevertheless, in coupled social‐ecological systems, the competition between vigorously pursued ecological programs and sustained economic demands can produce complex outcomes.

### 2.2 Modeling Approach and Experimental Design

In this study, we investigated how dominant land use changes driven by competing land use decisions, including ecological programs (the NFCP and GGP) and economic demands (cropland expansion and urbanization), influenced forest habitat quality in the CBMR, which served as a proxy for measuring biodiversity. The study utilized historical land use data from 1990 to 2020 and projected future land use changes for 2050 using the Conversion of Land Use and its Effects at Small Region Extent (CLUE‐S) model. The habitat quality changes from 1990 to 2050 were assessed using the Integrated Valuation of Ecosystem Services and Trade‐offs (InVEST) model, which considered factors such as habitat suitability, sensitivity, rarity, and threats. The relative contribution of these competing land use decisions to habitat quality changes was then explored using linear mixed‐effects models (LMMs).

#### 2.2.1 Projections of Land Use/Cover Change

Historical land use data for the years 1990, 1995, 2000, 2005, 2010, 2015, and 2020 at a 30 m resolution were obtained from the National Earth System Science Data Center (http://www.geodata.cn). The data contained six land use types: cropland, forest, grassland, wetland, built‐up land, and unused land.

Future climate data were assembled based on predictions from the CESM1‐BGC, CCSM4, CNRM‐CM5, FGOALS‐g2, MIROC5, and MRI‐CGCM3 global models under the high emission scenario (SSP 5–8.5), all of which were validated for applicability in the CBMR (Wang et al. 2019). The CLUE‐S model was employed to project future land use changes from 2020 to 2050 under the SSP 5–8.5 scenario in the CBMR (Wang et al. 2022). Four environmental characteristics were integrated into the future land use scenario: (1) contemporary land use patterns, (2) historical trends in land use changes, (3) the implementation of ecological programs (i.e., the NFCP and GGP), and (4) the needs of economic development and social well‐being. The CLUE‐S model projected future land uses by quantifying location suitability, setting spatial constraints, and determining conversion settings. Location suitability was assessed by quantifying the relationship between land use patterns and explanatory factors through logistic regression (Verburg et al. 2002). Land use variables were constrained by setting vital land use types as spatial constraints, and conversion settings were determined by the land use conversion matrix of the historical time series. A detailed description of the future land use projections was provided in the Supporting Information (Appendix A).

The model accuracy was assessed by comparing simulation results with observed data from satellite remote sensing images from 2000, 2010, and 2020, using the Kappa coefficient (van Vliet and Bregt 2011). Kappa coefficient values between 0.70 and 0.85 indicate good accuracy, while values greater than 0.85 represent the maximum agreement (Roy et al. 2024). The validation results showed that the kappa coefficients for 2000, 2010, and 2020 were 0.85, 0.88, and 0.88, respectively, indicating that the CLUE‐S simulation results aligned well with the observed trends, thereby confirming the model's reliability for further analysis.

#### 2.2.2 Assessment of Habitat Quality

We employed the habitat quality module of the InVEST model to assess spatial and temporal changes in habitat quality (Lei et al. 2022; Sun et al. 2019). The InVEST model has been widely used to assess the spatial dynamics of habitat responses to threats (Sun et al. 2019; Tang et al. 2020). The habitat quality module assessed the impacts of human activities on habitat quality by considering factors such as suitability, sensitivity, rarity, and threats (Wei et al. 2022; Zhao et al. 2022).

Model parameterization was performed by determining the habitat suitability, the maximum interference radius of threat factors, the weight of threat factors, and the relative sensitivity of each habitat type to threat factors (Huang et al. 2020; Wu et al. 2022). The suitability of different habitats was determined based on conditions in the study area and previous studies (Wei et al. 2022). Croplands and built‐up lands were selected as the major threat factors, and the associated parameters for these factors were determined in our study with reference to relevant studies (Wei et al. 2022; Wu et al. 2022; Zhao et al. 2022) (Table A1; Table A2). The InVEST model generated habitat quality values ranging from 0 to 1. We quantified habitat quality using the habitat quality index (HQI) and categorized it into three classes: low (0–0.2), medium (0.2–0.6), and high (0.6–1.0). A detailed description of the habitat quality assessment was provided in the Supporting Information (Appendix B).

### 2.3 Data Analysis

We estimated trends in forest habitat changes and their significance levels using the Mann–Kendall non‐parametric trend test from the “trend” package (Kondo et al. 2018; Thakur et al. 2021). LMMs were used to investigate the relative contributions of dominant land use conversions driven by ecological programs, cropland expansion, and urbanization to forest habitat quality changes at the sub‐watershed level (Qiu et al. 2020). The predictive variables were the proportions of the dominant land use conversions in each sub‐watershed (*n* = 98; Figure A1), including cropland expansion from forests and built‐up lands, urbanization from croplands and forests, forest restoration from croplands mainly driven by the GGP, unchanged forests mainly driven by the NFCP, and unchanged croplands, with “unchanged” referring to land use types that remained consistent. The response variable was the absolute value of the mean habitat quality change within each sub‐watershed, representing the varying degree of change, and it was log‐transformed for the subsequent analysis. Before conducting the analyses, collinearity among variables was first assessed using the mean Variance Inflation Factor (VIF) through the “Performance” package. Variables with VIF > 5 were excluded from further analyses to mitigate multicollinearity issues. An LMM was then constructed using restricted maximum likelihood (REML), with all six predictor variables included as fixed effects after standardization. To account for spatial autocorrelation, the sub‐watershed was incorporated as a random factor. A complete set of models, with all possible combinations of fixed effects, was generated to quantify the likelihood of each model using the “MuMIn” package (Posner et al. 2016). The coefficients and relative importance of each fixed variable were estimated through model averaging (Table A5), which considered model selection uncertainty and mitigated the potential influence of model misspecification (Schuldt et al. 2018). All analyses were conducted using R 4.1.3.

## 3 Results

### 3.1 Land Use Changes

**FIGURE 2:**
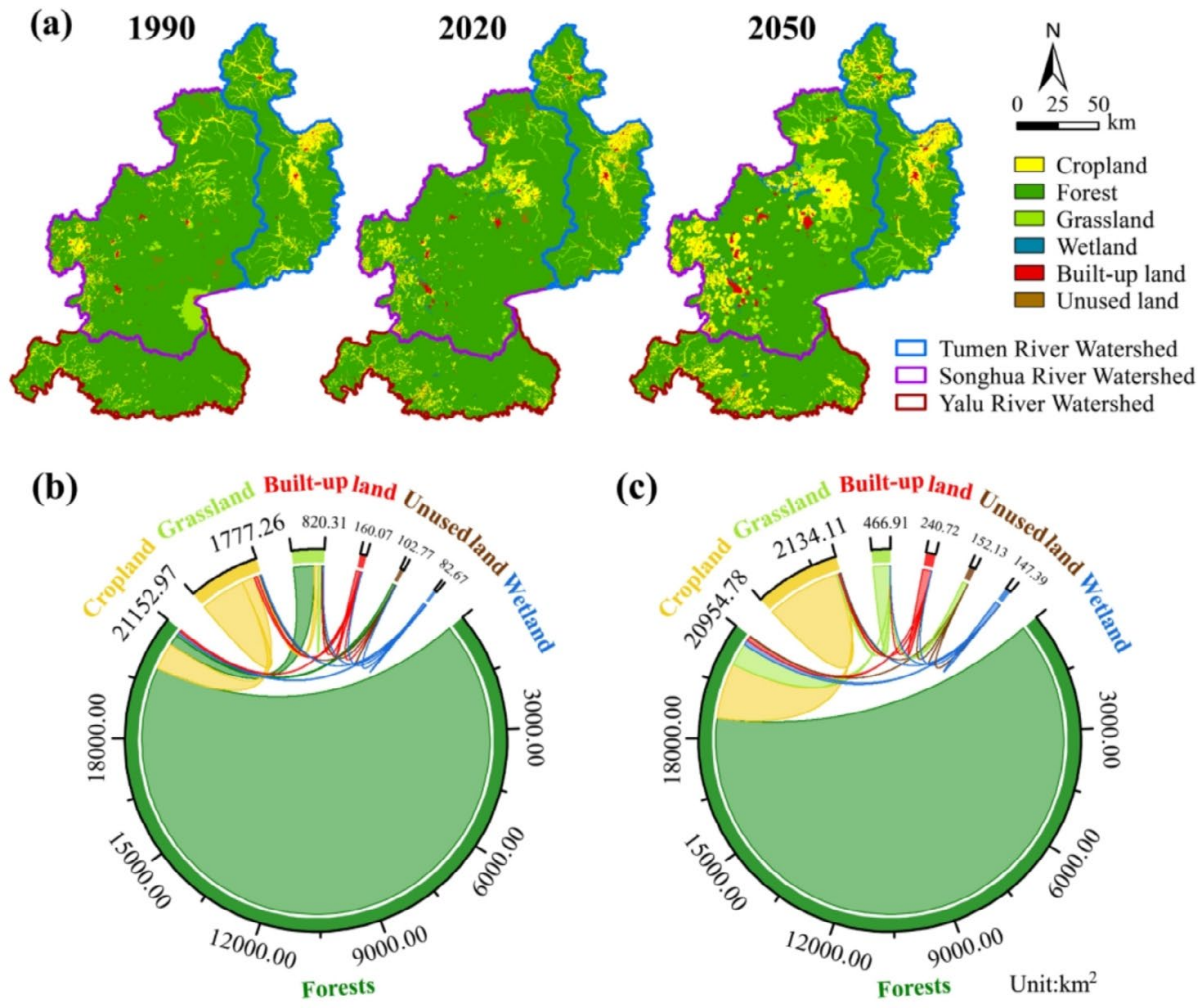
Land use changes in the CBMR: (a) land uses in 1990, 2020, and 2050; (b) land use changes from 1990 to 2020; (c) land use changes from 2020 to 2050.

During the period from 1990 to 2020, forests remained the dominant land use in the CBMR, comprising 87.79%–86.96% of the region, followed by croplands, grasslands, built‐up lands, unused lands, and wetlands (Figure 2; Figure A2). While most of the CBMR remained unchanged, 4.57% underwent significant shifts due to cropland expansion (+20.08%), urbanization (+50.39%), and deforestation (+0.93%). Croplands exhibited the largest increase, primarily due to the conversion of forests and grasslands. Although the NFCP and GGP programs converted large areas of croplands (337.07 km^2^) and grasslands (565.43 km^2^) into forests, many forests (729.57 km^2^) were lost to croplands (Table A3), leading to a slight net decrease in forest area. Wetlands (+78.66%) and unused lands (+48.04%) increased, while grasslands (−43.08%) decreased.

From 2020 to 2050, significant land use changes were projected, with forests expected to decline by 11.44%, primarily due to conversion to croplands (+64.84%), grasslands, wetlands, and built‐up areas. Notable increases were also observed in the areas of grasslands (+175.89%), built‐up lands (+69.10%), and wetlands (+103.06%). Additionally, large areas of unused lands (152.05 km^2^) were expected to be converted into grasslands, partially offsetting the significant grassland loss observed from 1990 to 2020 (Table A4).

### 3.2 Habitat Quality Changes

**FIGURE 3:**
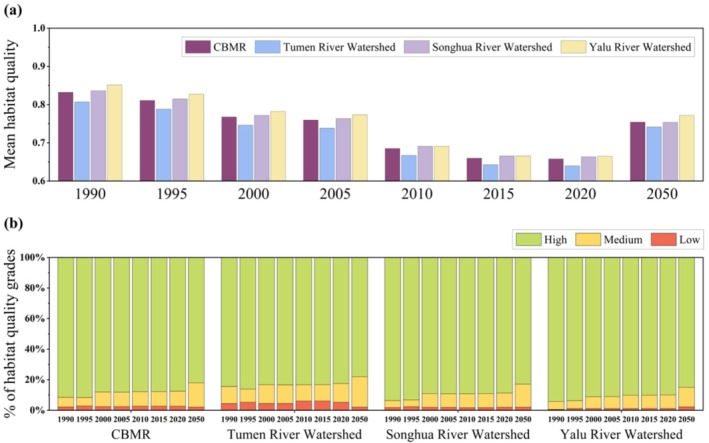
Changes in habitat quality from 1990 to 2050 for the entire CBMR and its three watersheds: (a) changes in the average habitat quality index; (b) changes in the proportion of high‐, medium‐, and low‐quality habitats.

**FIGURE 4:**
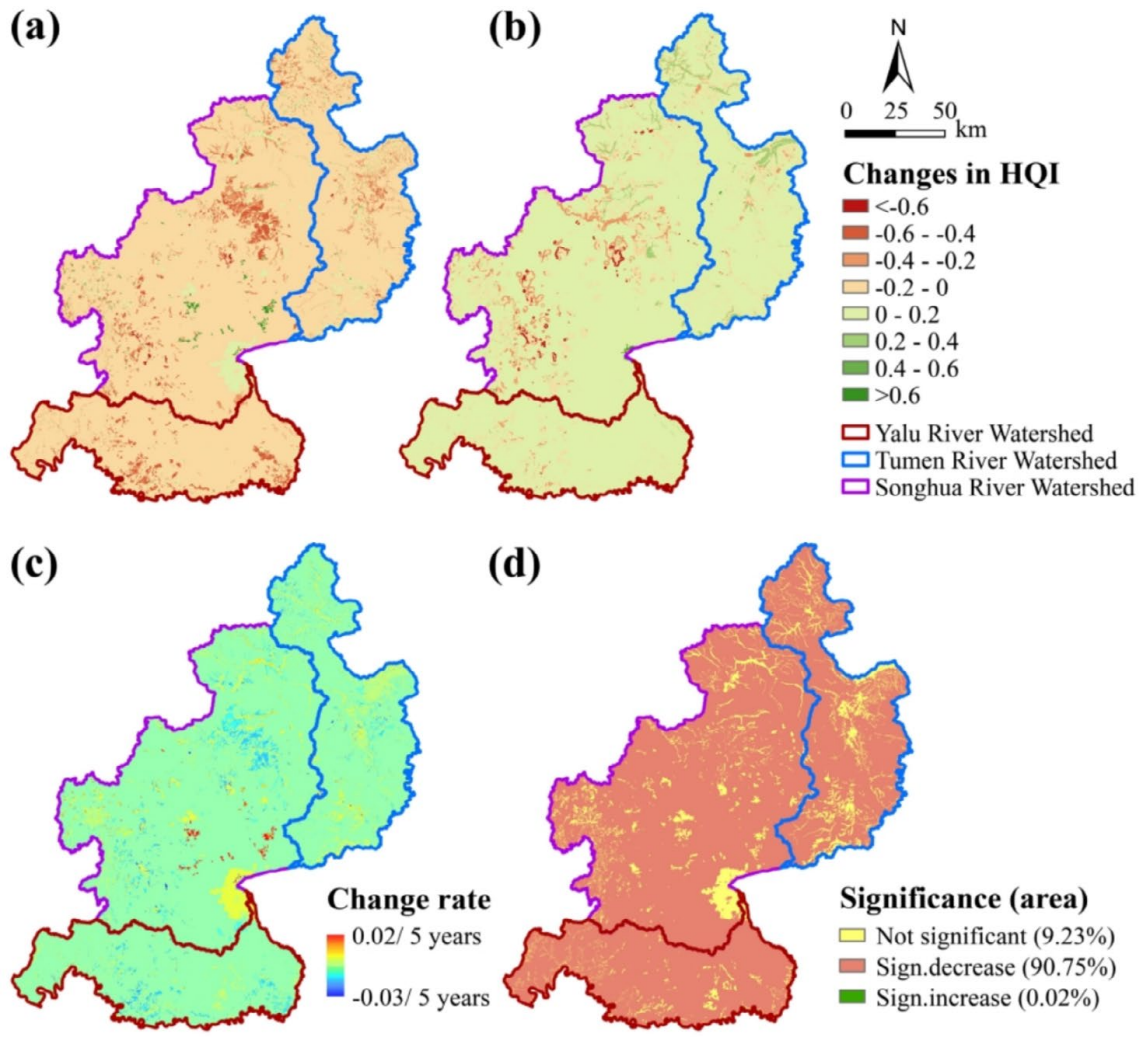
Spatial distribution of habitat quality changes in the CBMR: (a) changes in the habitat quality index (HQI) from 1990 to 2020; (b) changes in the HQI from 2020 to 2050; (c) trends in habitat quality changes from 1990 to 2020; (d) significance of habitat quality changes from 1990 to 2020.

**FIGURE 5:**
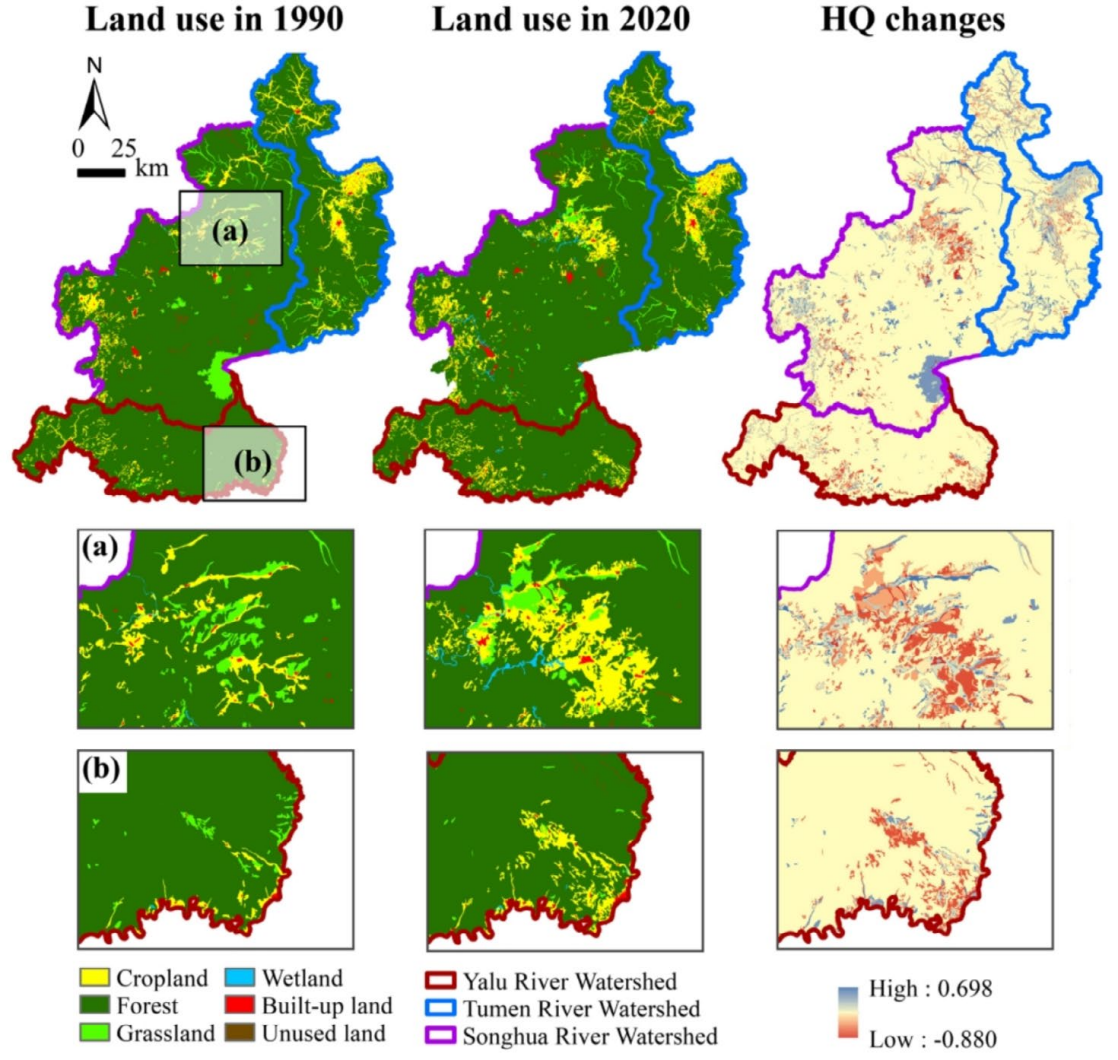
Selected areas with the most significant changes in land use and habitat quality from 1990 to 2020: (a) the north‐central part of the Songhua River Watershed; (b) the southern part of the Yalu River Watershed.

From 1990 to 2020, the average habitat quality across the region declined by 20.77%, with the mean HQI decreasing from 0.83 to 0.66 (Figure 3). A significant decline in HQI, ranging from 0 to 0.2, was observed in approximately 90.75% of the CBMR area (Figure 4d). The area of high‐quality habitats decreased from 91% to 87%, whereas medium‐ and low‐quality habitats increased from 6% to 10% and from 2% to 3%, respectively (Figure 3b). Habitat quality declined at a relatively rapid rate from 1990 to 2010, followed by a gradual deceleration from 2010 to 2020. At the watershed level, the overall declining trend in habitat quality was consistent across the three watersheds, albeit with considerable variations. Notably, the north‐central part of the Songhua River Watershed and the southern part of the Yalu River Watershed experienced the most substantial habitat declines, primarily attributed to cropland expansion and urbanization, occurring at a rate of −0.03 per 5‐year period (Figure 4; Figure 5). However, the southeastern Songhua River Watershed exhibited signs of habitat restoration, with an incremental rate of 0.02 per 5‐year period.

Projections for 2020–2050 showed an improvement in regional habitat quality, with the average HQI rising by 14.64%, from 0.66 to 0.75. The majority of the CBMR was expected to experience modest increases in HQI, primarily within the 0–0.2 range (Figure 4b). Despite the overall improvement, the proportion of high‐quality habitats decreased from 87% to 82%, while medium‐quality habitats increased from 10% to 16%. The Yalu River Watershed, with the highest quality habitat, was expected to experience the largest improvement (16.06%), followed by the Tumen River Watershed (15.87%). In contrast, habitat quality declines were expected to persist in the southwestern and north‐central parts of the Songhua River Watershed.

### 3.3 The Driving Factors

**FIGURE 6:**
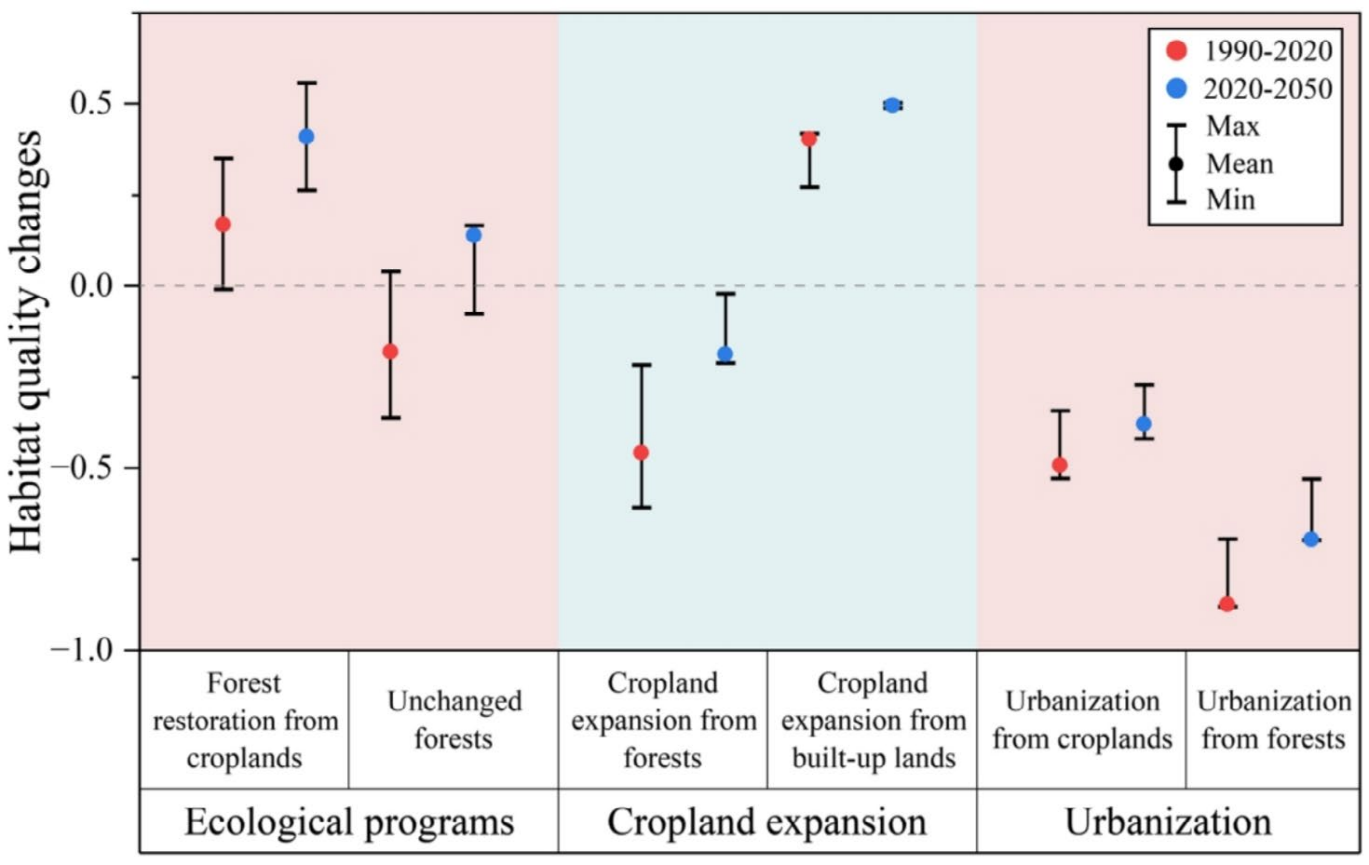
Changes in habitat quality driven by the dominant land use changes in the CBMR from 1990 to 2050.

**FIGURE 7:**
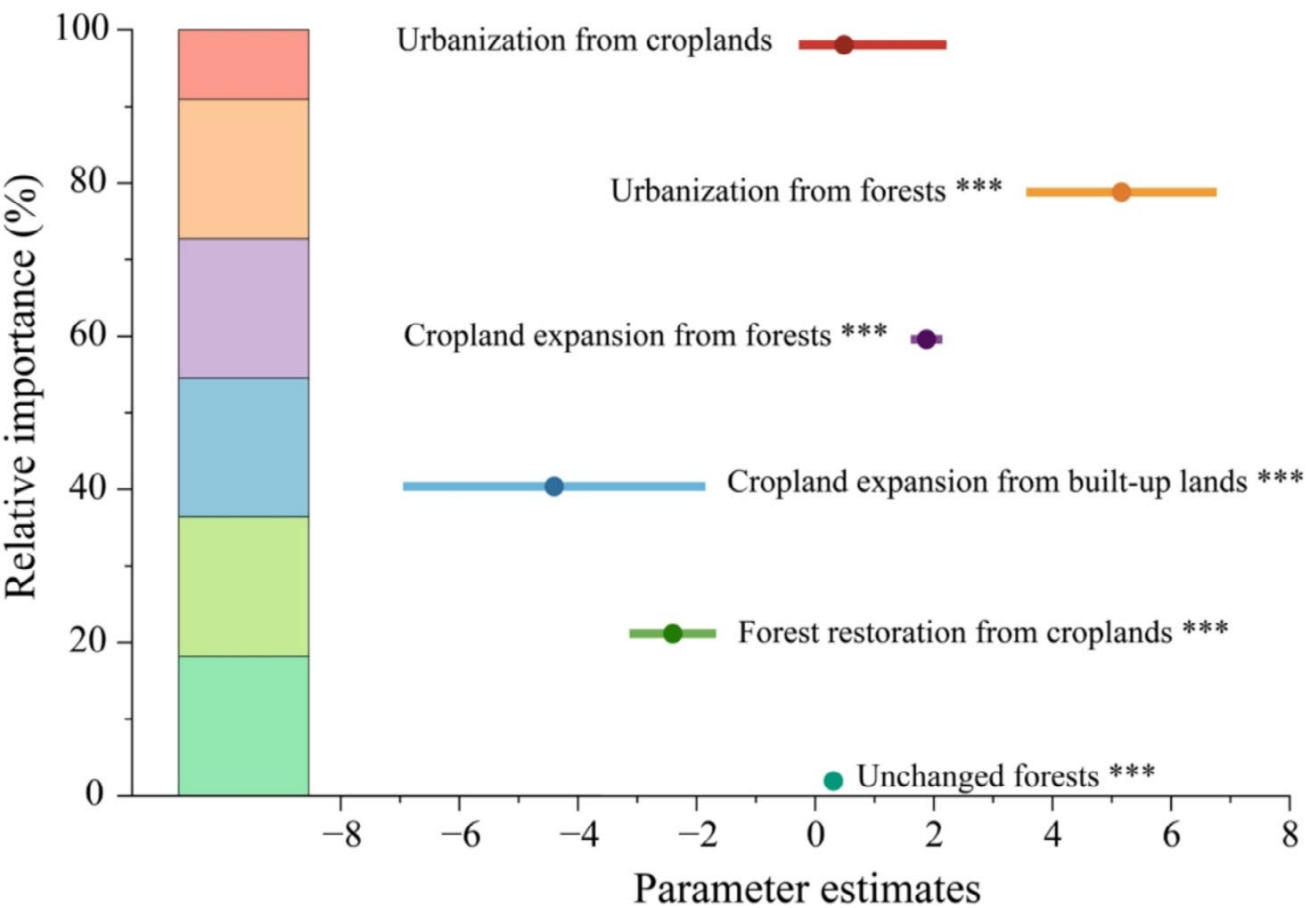
The relative importance of ecological programs (unchanged forests, forest restoration from croplands), cropland expansion (cropland expansion from built‐up lands, cropland expansion from forests), and urbanization (urbanization from forests, urbanization from croplands) on habitat quality changes (*p < 0.05; **p < 0.01; ***p < 0.001).

The LMM results revealed remarkably significant impacts of cropland expansion, urbanization, and ecological programs on habitat quality changes over the past 30 years (Table 1). The conservation of existing forests and forest restoration from croplands, primarily attributed to the NFCP and GGP, alongside cropland expansion and urbanization resulting from forest conversion, emerged as the most important drivers of habitat quality changes within the CBMR (*p* < 0.001, high relative importance) (Figure 7). Urbanization from forests (mean HQI: −0.88, coefficient: 5.164; *p* < 0.001) had the strongest negative effect on habitat quality, followed by cropland expansion into forests (mean HQI: −0.46; coefficient: 1.875; *p* < 0.001) (Figure 6). Conversely, cropland expansion into built‐up lands (mean HQI: +0.40; coefficient: −4.400; *p* < 0.001) and forest restoration from croplands (mean HQI: +0.26; coefficient: −2.401; *p* < 0.001) driven by the GGP, had positive impacts on habitat quality in this region. Interestingly, despite the NFCP's role in conserving existing forests, habitat quality still declined in these areas (mean HQI: −0.18; coefficient: 0.307; *p* < 0.001).

**TABLE 1:** Coefficients for the dominant land use changes as predictive variables, derived from model averaging, on the habitat quality changes in the CBMR from 1990 to 2020.

Response variable	Category	Predictors	Estimate	SE	*Z* value
The magnitude of HQ changes	Ecological programs	Unchanged forests	0.307	0.056	5.39
		Forest restoration from croplands	‐2.401	0.367	6.46
	Cropland expansion	Cropland expansion from built‐up lands	−4.400	1.284	3.38
		Cropland expansion from forests	1.875	0.134	13.78
	Urbanization	Urbanization from forests	5.164	0.809	6.30
		Urbanization from croplands	0.484	0.656	0.73

Abbreviations: HQ = habitat quality; SE = standard error.

## 4 Discussion

We assessed changes in temperate forest habitat quality and their driving factors, including multi‐land use decisions in the CBMR, Northeast China. Our results revealed a region‐wide decline in habitat quality over the past 30 years, with potential improvements projected under the future scenario. Cropland expansion and urbanization from forested areas had the most significant negative impacts, while ecological programs contributed positively to habitat quality. These findings highlight the importance of managing multi‐objective land use trade‐offs to sustain temperate forest habitats.

### 4.1 Spatial–Temporal Changes in Habitat Quality

Our results demonstrated region‐wide declines in forest habitat quality, with great variation observed across spatial and temporal scales. At the regional scale, habitat quality declined at a slower rate in the 20 years following the implementation of the NFCP and GGP, while projections indicated significant improvements over the next 30 years. However, at the sub‐regional scale, substantial improvement in habitat quality was observed within 10 years of the implementation of the NFCP and GGP in some areas, such as the southern part of the Songhua River Watershed (Figure A3). This finding highlights the significant variation in habitat quality changes across temperate forests owing to the high landscape heterogeneity and human activities, which further complicate the timeline of ecological restoration programs (Cosentino et al. 2014; Li et al. 2024; Uezu and Metzger 2016).

Our study also observed the most significant habitat quality declines in the Yalu River Watershed from 1990 to 2000, whereas this watershed exhibited the fastest recovery rate following the implementation of ecological programs. This finding suggests that primary forests with high‐value habitats in this region are more resilient to disturbances compared to secondary forests established through ecological initiatives (Gibson et al. 2011; Wang et al. 2023; Wang et al. 2019). This greater resilience is likely due to the higher species diversity and structural complexity of natural forests, which enhance ecosystem functions such as forest productivity, carbon sequestration, and water regulation, thereby enabling them to utilize resources more efficiently and recover from disturbances more effectively than planted forests (Nie et al. 2024; Paquette and Messier 2011; Yu et al. 2019). Therefore, the spatial heterogeneity and restoration rates driven by ecological programs with varying objectives in temperate forests warrant further investigation.

### 4.2 The Driving Factors of Habitat Quality Changes

The ecological programs improved habitat quality over long time scales by preserving existing forests and increasing forest cover in the CBMR, which was in line with recent studies (Sun et al. 2015; Yan et al. 2022; Zhang et al. 2023). Over the past 30 years, 94% of forests within the NFCP region remained intact, and reforested areas within the GGP region experienced an average habitat quality increase of 0.17. Overall, the GGP had more significant positive impacts on habitat quality improvements compared to the NFCP, although the NFCP played a crucial role in protecting forest habitats by reducing deforestation. This finding indicates variability in the benefits of ecological programs due to differences in land use targets and habitat conditions. Thus, ecological programs should be based on a comprehensive, multidimensional framework, as focusing on single‐factor effects (such as forest cover, short‐term benefits) may undermine the efforts of environmental protection (Birch et al. 2010; Cao et al. 2021). The effectiveness of ecological programs depends on the complex interactions of various natural and socioeconomic factors (Fu et al. 2018; Quan et al. 2023; Tong et al. 2017). Our results indicated that cropland expansion and urbanization from forests were the primary drivers of habitat quality declines, leading to both direct habitat loss and indirect negative effects on the habitat quality of unchanged forests. Cropland expansion from forests in the CBMR exceeded the area of forests gained from croplands by more than twofold (Table A3), offsetting the forest cover increase resulting from the GGP. Urbanization from forests led to the greatest declines in habitat quality, underscoring the necessity of strengthened implementation of the NFCP and controlling urban expansion within forested areas, especially in natural forests. Additionally, habitat quality declined even in some forests within the NFCP region, likely due to the negative impacts of widespread cropland expansion and urbanization on surrounding areas. The interplay between economic demands (e.g., cropland expansion and urbanization) and ecological policies contributes to large‐scale and high‐frequency land use conversions, which potentially undermine the overall effectiveness of ecological programs aimed at increasing forest cover and enhancing habitat quality (Dang et al. 2023; Ding et al. 2021). These findings highlight a potential trade‐off between economic development and ecological conservation in the CBMR, diminishing the effectiveness of ecological programs aimed at increasing forest habitat.

### 4.3 Management Implications

Our findings observed spatial heterogeneity in the effects of ecological programs on habitat quality, which may be attributed to variations in habitat conditions, such as species composition and available resources, as well as differences in the implementation of ecological programs. Additionally, ecological programs may experience time lags before generating positive ecological, social, and economic benefits (Cao et al. 2021; Zhou et al. 2022). Thus, appropriate ecological measures should consider spatiotemporal effects, targets and modes of implementation, and recovery speed to provide comprehensive results for the long‐term maximization of benefits. Furthermore, our study highlights a potential trade‐off between economic development (cropland expansion and urbanization) and ecological conservation, which may diminish the effectiveness of ecological programs. The role of competing land uses remains underappreciated, particularly the consequences of the interconversion of forests with other land use types for economic and social purposes (Meli et al. 2024). Our findings suggest that future management practices should manage construction intensity and population density in alignment with environmental carrying capacity to achieve the win‐win goal of protecting forest habitats while promoting regional social and economic development.

The complex interplay of multi‐land use decisions amplifies the challenges of ecological management and increases the unpredictability of ecological outcomes, especially in the context of climate change (Fu et al. 2024; Shen et al. 2023). Climate change can directly impact species survival or indirectly alter habitat conditions, such as range, temperature, and habitat quality (Mondal et al. 2022). However, studies suggest that the adverse effects of climate change can be mitigated or adapted to by implementing appropriate management strategies (Li, Li et al. 2023; Yu et al. 2020), even in urbanized areas and agricultural lands (Choi et al. 2021). This necessitates a comprehensive approach that considers the complex ecological processes driven by multi‐land use decisions and develops a long‐term dynamic habitat model within a social‐ecological framework to inform management strategies under ongoing climate change.

### 4.4 Limitations

Some factors not considered in this study may contribute to uncertainty in our results. While we incorporated the dominant land use changes as the primary driving factors of habitat loss and degradation in this region, other land use conversions (e.g., forest restoration from grasslands) were not included in the analysis and might also influence regional habitat quality, albeit modestly (Zhang et al. 2024). Furthermore, uncertainty in future climate projections may influence the CLUE‐S model results (Kiziridis et al. 2023). We derived climate change data using ensemble methods to enhance the robustness of our projections and validated the land use simulation results from the CLUE‐S model to ensure their applicability and reliability (Wang et al. 2022; Wang et al. 2019). Additionally, although the InVEST model has been widely recognized as a reliable tool for assessing biodiversity and habitat quality dynamics (Upadhaya and Dwivedi 2019), it primarily focuses on the impacts of land use changes on habitat loss and stress without fully capturing complex ecosystem processes and species‐specific variations in stress sensitivity (Moreira et al. 2018). Hence, future studies should improve habitat quality assessment by employing a more integrative modeling approach that integrates ecological interactions and land use planning, enabling a balanced trade‐off between social needs and biodiversity conservation.

## 5 Conclusions

This study assessed the impacts of land use changes driven by competing land use decisions, including ecological programs and economic demands (cropland expansion and urbanization), on temperate forest habitat quality in the CBMR, Northeast China. Our results revealed widespread declines in habitat quality across the region over the past three decades, with projected improvements under the future scenario. Ecological programs contributed to long‐term habitat improvements by preserving and increasing forest cover. However, cropland expansion and urbanization from forests were identified as the primary drivers of habitat quality degradation, leading to both direct habitat loss and indirect negative effects on the quality of unchanged forests. Our results suggest that the trade‐offs between ecological conservation and economic demands in temperate forests, in turn, influence the effectiveness of ecological initiatives. These findings highlight the importance of considering the complex interactions between competing land use decisions, with the goal of balancing ecological and socio‐economic objectives to achieve sustainable outcomes.

## Author Contributions

**Li Liu:** data curation (equal), formal analysis (equal), investigation (equal), methodology (equal), software (equal), validation (equal), visualization (equal), writing – original draft (equal), writing – review and editing (equal). **Wen J. Wang:** conceptualization (equal), funding acquisition (equal), methodology (equal), project administration (equal), supervision (equal), validation (equal), writing – original draft (equal), writing – review and editing (equal). **Lei Wang:** investigation (equal), visualization (equal). **Yu Cong:** validation (equal), writing – review and editing (equal). **Haitao Wu:** funding acquisition (equal), validation (equal), writing – review and editing (equal).

## Conflicts of Interest

The authors declare no conflicts of interest.

## Supporting information


Appendix S1.

## Data Availability

The relevant data of this paper can be found in the Supporting Information and were also accessible through Figshare (https://figshare.com/s/b9fd42a7b75e3bebeb79).
