# Supplementary material for: Impacts of Multi‐Land Use Decisions on Temperate Forest Habitat Quality in the Changbai Mountain Region, Northeast China
Source: Ecol Evol. 2025 Mar 27;15(4):e71123. doi: 10.1002/ece3.71123 (PMC11949574; doi:10.1002/ece3.71123)
Supplement: Supplementary file 1 — Appendix S1. [file ECE3-15-e71123-s001.docx]

**Supplementary Materials**

**Impacts of multi-land use decisions on temperate forest habitat quality in the** **Changbai Mountain region, Northeast China**

Li Liu^1,2^, Wen J. Wang^1,^* , Lei Wang^1^, Yu Cong^1^, Haitao Wu^1^

^1.^ Northeast Institute of Geography and Agroecology, Chinese Academy of Sciences, Changchun 130102, China

^2.^ University of Chinese Academy of Sciences, Beijing 100049, China

**Corresponding author:** Wen J. Wang: [wangwenj@iga.ac.cn](mailto:wangwenj@iga.ac.cn)

**Appendix A.** **Details of the simulation of future land use using the CLUE-S model**

This study employed the Conversion of Land Use and its Effects at Small Region Extent (CLUE-S) model to project land use changes from 2020 to 2050 in the Changbai Mountain region (Wang et al., 2022). To assess habitat conditions and ecological potential in temperate forests under a worst-case scenario, and to derive adaptive forest management strategies to cope with climate change, we conducted future land use simulations under the SSP 5-8.5 climate scenario. Future climate data were obtained from the Coupled Model Intercomparison Project (Phase 6). We assembled daily precipitation, minimum and maximum temperature, and radiation data, then regionalized these to 1 km resolution for the CBMR using the WFR-ARW regional dynamical model. The CLUE-S model is a spatially explicit land use model designed to simulate the dynamic competition between different land use types, and has demonstrated adaptability and accuracy in simulating land dynamics at small and medium spatial scales. We assessed positional suitability by quantifying the relationship between land use patterns and explanatory factors through logistic regression (Verburg et al., 2009). We employed a traditional logistic stepwise regression method to predict the competitiveness of various land use types for each raster cell, and assessed the probability of the presence of a particular land use type at each raster cell (Verburg et al., 2002). This probability contributed to determining the regional suitability of each raster cell for a specific land use type. The formula of the model is as follows:

$\log\left( \frac{P_{i}}{1-P_{i}} \right)=\beta_{0}+\beta_{1}X_{1i}+\beta_{2}X_{2i}+\cdots+\beta_{n}X_{ni}$ (A.1)

Where *Pi* represents the probability of land use type *i* occurring in the raster cell; *X* represents the driving factors affecting the occurrence of this probability; *β* represents the regression coefficients of different driving factors. Land use variables were constrained by designating forests and wetlands as spatial constraints. The conversion settings were determined based on the land use conversion matrix of the past time series, which defines the historical patterns of land use change.

This study validated the model results by comparing the simulation results with satellite remote sensing data for the years 2000, 2010, and 2020 using the Kappa index. The Kappa index is a widely used metric to assess the agreement between simulated land cover and actual land cover derived from remote sensing data. The Kappa values for 2000, 2010, and 2020 were 0.85, 0.88, and 0.88, respectively, indicating that the CLUE-S simulation results aligned well with the observed trends.

**References:**

Verburg, P. H., & Overmars, K. P. 2009. Combining top-down and bottom-up dynamics in land use modeling: Exploring the future of abandoned farmlands in Europe with the dyna-clue model. *Landscape Ecology*, 24(9), 1167-1181.

Verburg, P. H., et al. 2002. Modeling the spatial dynamics of regional land use: The clue-s model. *Environmental Management*, 30(3), 391-405.

Wang, H., et al. 2022. Impacts of future climate and land use/cover changes on water-related ecosystem services in Changbai Mountains, Northeast China. Frontiers in *Ecology and Evolution*, 10.

**Appendix B. Details of the assessment of habitat quality using the InVEST model**

This study employed the habitat quality module in the Integrated Valuation of Ecosystem Services and Trade-offs (InVEST) model to quantitatively assess habitat quality and reflect the biodiversity status of the forest ecosystem of the CBMR. The InVEST model is one of the most important models for assessing the functioning of ecosystem services (Tang, et al. 2020). The habitat quality module assesses the potential of ecosystems to support species survival and reproduction by calculating the degree of habitat degradation to assess regional habitat quality (Zhao, et al. 2022). The formula for calculating habitat quality is as follows:

$Q_{xj}=H_{j}\left[ 1-\left( \frac{D_{xj}^{z}}{D_{xj}^{z}+k^{2}} \right) \right]$ (A.2)

In Eq. (A.2), $Q_{xj}$ represents the habitat quality index of grid *x* in land use type *j*; $H_{j}$ represents the suitability of land use type *j* as habitat; $D_{xj}^{z}$ represents the habitat degradation degree of grid *x* in land use type *j*; *z* represents the default parameter of the model, and *k* represents the semi-saturation constant, typically set to 0.5.

$D_{xj}=\Sigma_{r=1}^{R}\Sigma_{y=1}^{\gamma_{r}}\left( \omega_{r}/\sum_{r=1}^{R} \omega_{r} \right)r_{y}i_{rxy}\beta_{x}S_{jr}$ (A.3)

$i_{rxy}=1-\left( \frac{d_{xy}}{d_{rmax}} \right)if linear$ (A.4)

$i_{rxy}=exp\left( -\left( \frac{2.99}{d_{rmax}} \right)d_{xy} \right)if exponential$ (A.5)

In Eqs. (A.3), (A.4), (A.5), *R* represents the number of threat factors, $\gamma_{r}$ represents the set of grids in the *r* threat factor layer, $\omega_{r}$ represents the weight of threat factor *r*, $r_{y}$ represents the stress value of grid *y*, $\beta_{x}$ represents the accessibility of grid *x*, $S_{jr}$ represents the relative sensitivity of habitat quality of each land use type to threat factors, $i_{rxy}$ represents the influence of threat Factor *r* on grid *y* in the habitat of grid *x*, $d_{xy}$ represents the linear distance between grid *x* and grid *y*, and $d_{rmax}$ represents the maximum interference radius of threat Factor *r*.

Based on conditions in the study area and previous studies (Wu, et al. 2022,Wei, et al. 2022), we determined the habitat suitability and the relevant parameters for each threat factor in our study, as shown in Table A1 and A2.

**Table A1.** Attributes of the threat factors for the InVEST Habitat Quality module.

| Maximum distance | Weight | Threats | Spatial decay type |
| --- | --- | --- | --- |
| 1 | 0.70 | Cropland | Linear |
| 8 | 1 | Built-up land | Exponential |

**Table A2.** Habitat suitability and sensitivity of land use types to each threat factor.

| Land use type | Habitat suitability | Cropland | Built-up land |
| --- | --- | --- | --- |
| Cropland | 0 | 0 | 0 |
| Forest | 1 | 0.70 | 0.80 |
| Grassland | 1 | 0.70 | 0.80 |
| Wetland | 1 | 0.70 | 0.80 |

**References:**

Tang, F., et al. 2020. Land-use change in Changli County, China: Predicting its spatio-temporal evolution in habitat quality. *Ecological Indicators*, 117, 106719.

Wu, J., et al. 2022. Projections of land use change and habitat quality assessment by coupling climate change and development patterns. *Science of The Total Environment*, 847, 157491.

Wei, Q., et al. 2022. Temporal and spatial variation analysis of habitat quality on the PLUS-InVEST model for Ebinur Lake Basin, China. *Ecological Indicators*, 145, 109632.

Zhao, Y., et al. 2022. Effects of human activity intensity on habitat quality based on nighttime light remote sensing: A case study of Northern Shaanxi, China. *Science of The Total Environment*, 851, 158037.

**Appendix C. Additional figures and tables**


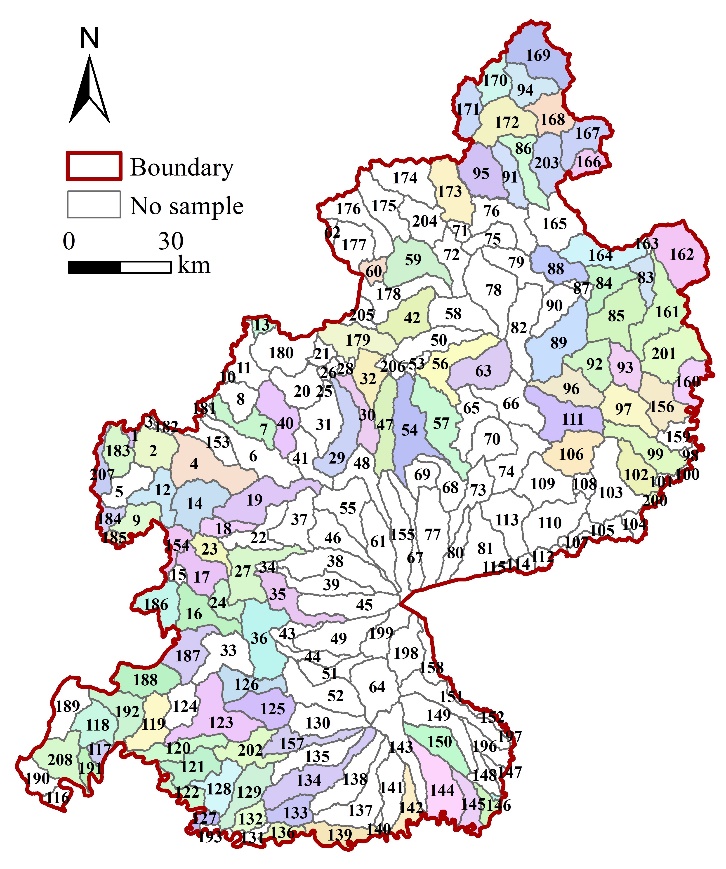


**Fig. A1.** Spatial distribution of the sub-watersheds (n=98) included in the analysis using linear mixed-effects models.


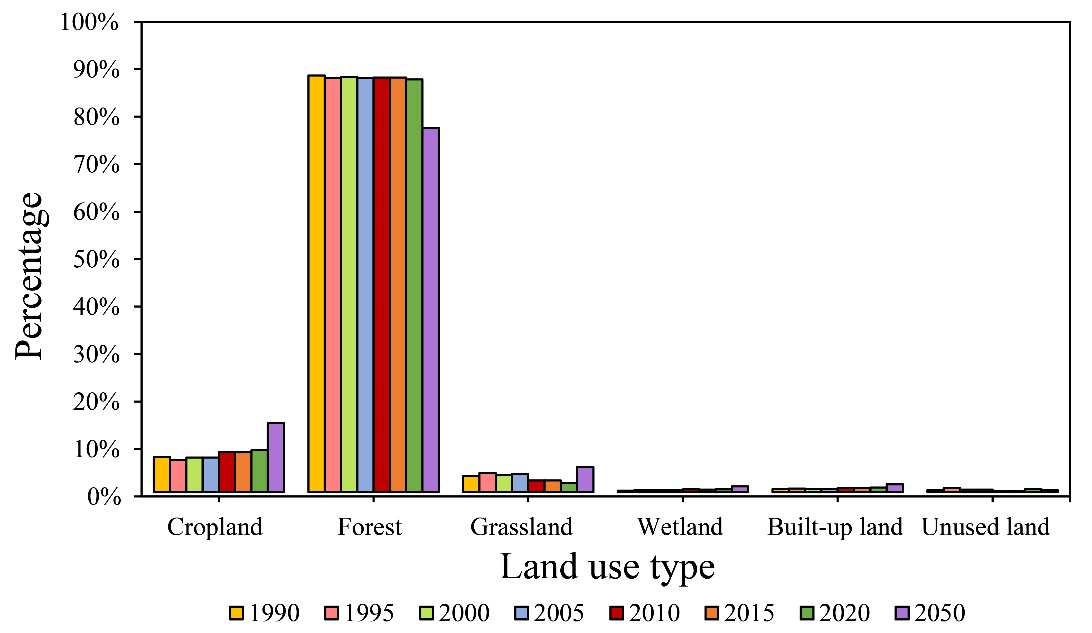


**Fig. A2.** Changes in the proportions of land use types in the Changbai Mountain region (CBMR) from 1990 to 2050.


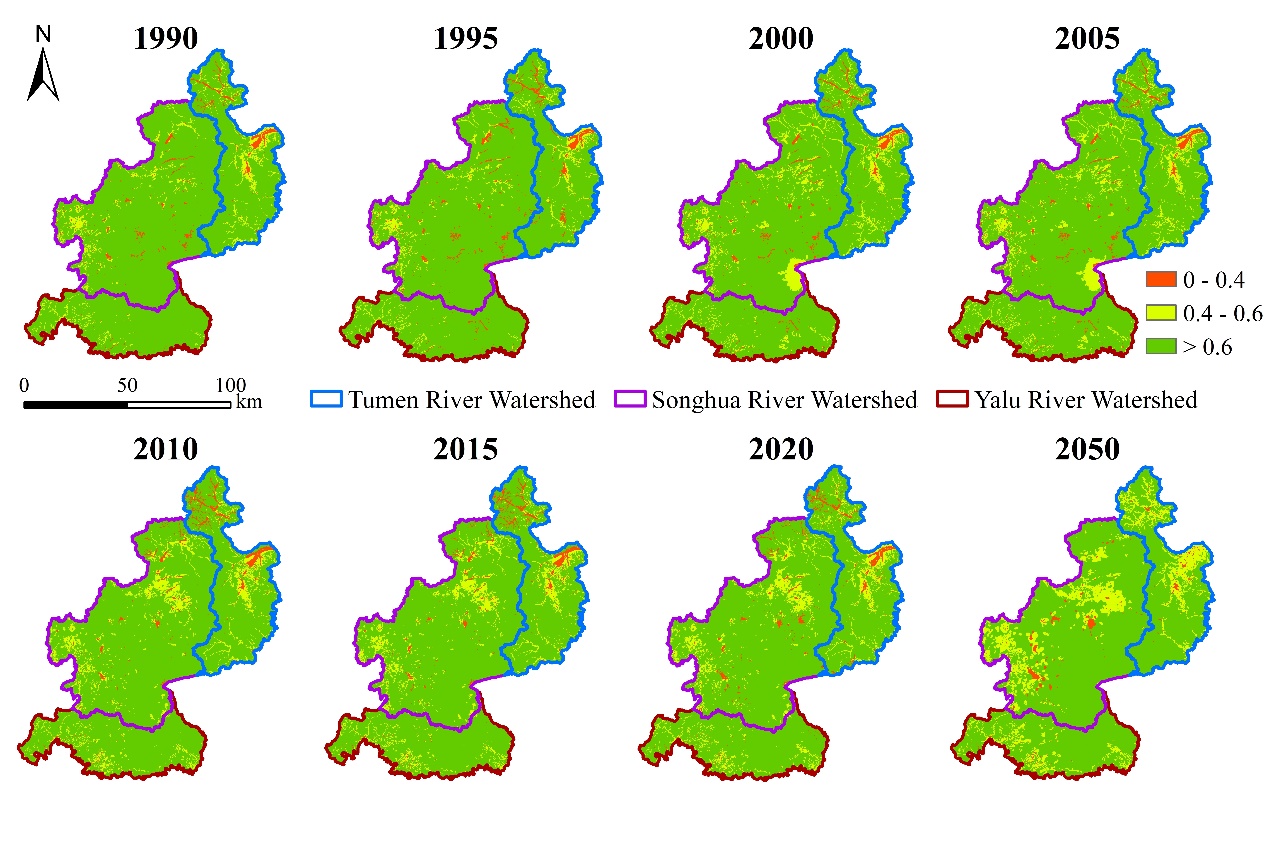


**Fig. A3.** Spatial distribution of habitat quality in the CBMR from 1990 to 2050.

**Table A3.** Land use conversion matrix from 1990 to 2020.

|  | | Land use types in 2020 (km^2^) | | | | | | |
| --- | --- | --- | --- | --- | --- | --- | --- | --- |
|  |  | Cropland | Forest | Grassland | Wetland | Built-up land | Unused  land | Total |
| Land use types in 1990 (km^2^) | Cropland | 1,228.49 | 337.07 | 79.2 | 19.12 | 88.57 | 24.22 | 1,776.66 |
|  | Forest | 729.57 | 19,949.70 | 290.6 | 64.6 | 47.58 | 71.08 | 21,153.13 |
|  | Grassland | 116.49 | 565.43 | 90.08 | 2.27 | 7.34 | 38.34 | 819.95 |
|  | Wetland | 4.48 | 17.37 | 1.56 | 58.66 | 0.33 | 0.00 | 82.39 |
|  | Built-up land | 50.22 | 11.62 | 1.60 | 0.57 | 95.36 | 0.06 | 159.43 |
|  | Unused land | 4.56 | 74.98 | 3.63 | 0.43 | 0.75 | 18.36 | 102.72 |
|  | Total | 2,133.80 | 20,956.17 | 466.68 | 145.64 | 239.92 | 152.07 | 24,094.28 |

**Table A4.** Land use conversion matrix from 2020 to 2050.

|  | | Land use types in 2050 (km^2^) | | | | | | |
| --- | --- | --- | --- | --- | --- | --- | --- | --- |
|  |  | Cropland | Forest | Grassland | Wetland | Built-up land | Unused  land | Total |
| Land use types in 2020 (km^2^) | Cropland | 2,004.33 | 0.59 | 62.55 | 18.63 | 45.99 | 2.02 | 2,134.11 |
|  | Forest | 1,510.66 | 18,556.23 | 609.19 | 140.62 | 122.35 | 15.74 | 20,954.78 |
|  | Grassland | 0.22 | 1.22 | 464.28 | 0.00 | 1.08 | 0.11 | 466.91 |
|  | Wetland | 0.00 | 0.00 | 0.00 | 140.66 | 0.00 | 6.73 | 147.39 |
|  | Built-up land | 2.74 | 0.13 | 0.11 | 0.01 | 237.65 | 0.07 | 240.72 |
|  | Unused land | 0.00 | 0.02 | 152.05 | 0.01 | 0.01 | 0.05 | 152.13 |
|  | Total | 3,517.95 | 18,558.18 | 1,288.18 | 299.93 | 407.07 | 24.73 | 24,096.04 |

**Table A5.** Coefficients for the averaged models. The intercept, degrees of freedom (df), AICc, AICc difference (ΔAICc), and Akaike weights derived from the AICc (AICc-W) for each model are provided.

| Model | Intercept | df | AICc | △AICc | AICc-W |
| --- | --- | --- | --- | --- | --- |
| 62nd | -1.987 | 8 | -303.9 | 0.00 | 0.501 |
| 64th | -2.019 | 9 | -303.9 | 0.010 | 0.499 |
